# New insights into metabolism dysregulation after TBI

**DOI:** 10.1186/s12974-024-03177-6

**Published:** 2024-07-29

**Authors:** Helena C. Oft, Dennis W. Simon, Dandan Sun

**Affiliations:** 1 Department of Neurology, University of Pittsburgh, 3501 Fifth Avenue, Pittsburgh, PA 15213 USA; 2 Pittsburgh Institute for Neurodegenerative Disorders, University of Pittsburgh, Pittsburgh, PA 15213 USA; 3 Department of Critical Care Medicine, School of Medicine, University of Pittsburgh, 4401 Penn Avenue, Pittsburgh, PA 15224 USA; 4 Department of Pediatrics, University of Pittsburgh School of Medicine, Pittsburgh, PA USA; 5 Safar Center for Resuscitation Research, University of Pittsburgh School of Medicine, Pittsburgh, PA USA; 6 Children’s Neuroscience Institute, Children’s Hospital of Pittsburgh, Pittsburgh, PA USA; 7 Geriatric Research, Educational and Clinical Center, Veterans Affairs Pittsburgh Health Care System, Pittsburgh, PA 15213 USA

**Keywords:** Glucose metabolism, Gut-brain axis, Ketometabolism, Lipid oxidation, Mitochondrial metabolism, Microbiome

## Abstract

Traumatic brain injury (TBI) remains a leading cause of death and disability that places a great physical, social, and financial burden on individuals and the health system. In this review, we summarize new research into the metabolic changes described in clinical TBI trials, some of which have already shown promise for informing injury classification and staging. We focus our discussion on derangements in glucose metabolism, cell respiration/mitochondrial function and changes to ketone and lipid metabolism/oxidation to emphasize potentially novel biomarkers for clinical outcome prediction and intervention and offer new insights into possible underlying mechanisms from preclinical research of TBI pathology. Finally, we discuss nutrition supplementation studies that aim to harness the gut/microbiome-brain connection and manipulate systemic/cellular metabolism to improve post-TBI recovery. Taken together, this narrative review summarizes published TBI-associated changes in glucose and lipid metabolism, highlighting potential metabolite biomarkers for clinical use, the cellular processes linking these markers to TBI pathology as well as the limitations and future considerations for TBI “omics” work.

## Overview of traumatic brain injury management and limited therapies

In 2015, the United States Center for Disease Control (CDC) delivered its first report on traumatic brain injury (TBI) to the United States Congress, citing critical challenges related to injury detection and treatment [1]. Preventative measures, such as infrastructure improvements that reduce motor vehicle accidents, have contributed to decreases in TBI worldwide. The most recent Global Burden of Diseases, Injuries, and Risk Factors (GBD) study reported an age-standardized prevalence rate of 448.6 per 100,000 people in 2021 compared to the 2019 edition’s cited rate of 599 cases per 100,000 people [2–4]. However, these numbers do not capture the extent to which TBI affects those who do not seek immediate medical attention, those with masking comorbidities or poly-trauma and those with unclear mechanisms of injury [5]. The CDC estimates that between 3.2–5.3 million people in the US are living with a TBI-related disability that can include pain, cognitive, emotional and motor disturbances and secondary neurological disorders such as posttraumatic epilepsy and mood disorders, dementia syndromes, chronic traumatic encephalopathy, etc. [1, 6]. The GBD estimated that TBI resulted in 5.490 million years lived with disability across surveyed countries in 2021 alone [3]. In addition to the physical cost for patients, those diagnosed with nonfatal TBI in the US may accumulate an average of $19,598 per person annually in additional healthcare costs [7]. Moreover, these burdens are not distributed equally; vulnerable populations, unhoused or incarcerated individuals, marginalized racial and ethnic groups, victims of interpersonal or combat violence, young children or older adults are at greater risk for TBI and associated disability. They also experience unique barriers to TBI detection and care [5, 8, 9]. Since the CDC’s TBI report to the US Congress, federal agencies have invested more than two billion dollars in improving TBI research and treatment aimed at addressing this global priority [10].

Despite the proliferation of research that has expanded our understanding of TBI epidemiology and pathogenesis, the updated consensus for clinical management in the hospital setting does not define reliable biomarkers or drug therapies aimed at directly limiting secondary brain injury [11, 12]. Briefly, patients with TBI exposure are triaged based on clinical factors including the presence of other life-threatening injuries, neurologic exam (as assessed by Glasgow Coma Scale, GCS) and neuroimaging. In addition to routine medical management, monitoring of glucose and/or lactate levels has been described in some TBI protocols [5, 11–13]. Patients may receive a CT scan according to the center’s policy, and though this decision-making is typically guided by the New Orleans Criteria or Canadian Head CT rules, they may vary across clinical sites [14]. Precise clinical decision-making remains challenging despite recent advances informed by data from multisite observational studies including TRACK-TBI [15–17], CENTER-TBI [18, 19] and the CREACTIVE project [20] to name a few. These challenges highlight a need for new research aimed at better understanding TBI pathogenesis and identifying specific biomarkers corresponding to underlying cellular, structural and secretomic changes that can improve clinical decision making. In this review, we focus on the body of clinical and preclinical research aimed at identifying such TBI-induced alterations in cellular metabolism. Emphasizing the overlap and differences between bench and bedside research findings, we examine the translational potential of proposed metabolic biomarkers for improving TBI management and as therapeutic targets.

## Blood and brain biomarkers for TBI-mediated changes in metabolism

Differences in TBI management protocols and a lack of drug options to target underlying TBI pathology demonstrate a need for biomarkers that can improve diagnosis and prognosis determination for patients with TBI. The utility of putative blood biomarkers, including glial fibrillary acidic protein (GFAP), ubiquitin carboxy-terminal hydrolase L1 (UCH-L1), S100 calcium-binding protein B (S100B) and Neurofilament-light (NF-L), has been evaluated by multicenter studies such as ALERT, CENTER-TBI, TRACK-TBI and BIO-AX-TBI [5, 21]. Among these candidates, GFAP most consistently predicts CT anomalies, and to date, only GFAP/UCH-L1 testing has been approved for clinical use by the FDA [16, 20, 22]. However, these tests have limitations, including nonspecific readings in the context of polytrauma, limited availability in the acute care setting and minimal mechanistic insight [23, 24]. This led to expanded research on the contributions of metabolic dysfunction to TBI in the last decade using blood samples and cerebral microdialysis (CMD) [25]. Microdialysis utilizes a catheter and a semipermeable membrane inserted into the brain parenchyma (frequently in the frontal lobe, proximal to existing ICP or PbtO2 monitors) to detect changes in brain metabolites with high temporal and spatial resolution [26]. This precision microdialytic sampling may provide more insight into the mechanisms underlying TBI pathogenesis and secondary injury than systemic indicators in blood or cerebrospinal fluid (CSF). CMD may be especially useful in cases where invasive neuromonitoring of the ICP is already needed. Nuclear magnetic resonance and mass spectrometry with gas or liquid chromatography are the most commonly used methods for analyzing CMD samples [27]. Reviews of the growing CMD data pool have suggested the importance of including cellular metabolism markers in TBI classification and prognosis [28]. The most recent consensus statement from the International Microdialysis Forum, released in 2015, summarized key advances in the CMD clinical usage and contributions to neurocritical care since 2004 [25]. Although continuous monitoring with CMD might allow for greater temporal resolution, there are still limitations. CMD studies are more invasive than serum collection, limiting large scale sampling, generalizability of conclusions and increasing the impact of study design discrepancies (sample collection times, outcome follow-up, etc.) on reported values [28]. More so than in serum sampling, parenchymal fluid samples are small in volume, perhaps explaining why the published literature has not explored as comprehensive a metabolite profile as is reported in serum metabolomics work. As CMD collects local parenchymal fluid, interpatient variation and minor regional differences in probe placement (to the site of primary injury) can also increase the sample size required to achieve statistical significance [29]. The following sections highlight key metabolites implicated by several studies as putative high-yield targets for CMD/serum detection or therapeutic intervention.

### Glucose

In the last few years, “omics” analyses in larger multicenter studies, such as CENTER-TBI, CanTBI, and TBI-Care, have significantly expanded our understanding of the metabolic landscape after TBI. Glucose metabolism substrates and metabolites are among the best characterized biomarkers in these studies. Researchers have sought to identify specific patterns among these variables corresponding to specific disease etiology, pathogenesis, and prognosis [18, 24, 28, 30–33]. These signatures or “fingerprints” vary across trials but demonstrate the utility of metabolomics for prognostication as well as certain overarching traits when individual metabolites do not reach significance. Åkerlund et al.’s analysis of 1728 patients with TBI enrolled in the CENTER-TBI study during their intensive care unit (ICU) stay identified clusters of patients with lower GCSs and a “deranged metabolism” pattern defined by acidosis, elevated lactate and glucose [18]. They found that metabolic derangements were associated with increased risk of mortality in both moderate and severe TBI clusters. Generally, the detection of increased serum levels of sugar derivatives, including gluconates [30], pyruvate, lactate, d-( +)-galacturonic acid and (myo-)inositol [31], 2,3-bisphosphoglyceric acid (2,3-BPG) and pentitol-3-desoxy [34], particularly in the first 24 h after TBI, corresponded to poorer clinical outcomes and increased mortality (Table 1). Although these analyses are not specific to the central nervous system (CNS), they do offer clues to ongoing brain injury pathogenesis, as the increased permeability of the blood–brain barrier (BBB) after TBI allows for greater exchange of CNS/peripheral solutes, and many of these metabolites are also detected in the brain parenchyma [34]. For instance, Banoei et al. reported that more extreme changes in serum metabolite profiles from days 1 to 4 after severe TBI corresponded to unfavorable clinical outcomes and that serum metabolites on day 4 could better predict outcomes than serum metabolites collected on day 1 [27]. They suggested that this alludes to the impact of secondary injuries on metabolism over the days after injury but called for additional studies to expand the data to additional timepoints [27]. A smaller pilot study also revealed that cerebral glucose delivery was determined by plasma glucose concentration and cerebral blood flow, with injured brains exhibiting less glucose uptake and utilization around lesions but increased utilization at distal hyperglycolytic sites in the brain [32]. This overall reduction in glucose utilization detected by 18F-fluorodeoxyglucose-PET (positron emission tomography) imaging persists in the context of chronic cognitive impairment after TBI [35]. However, in studies exploring TBI in polytrauma patients, serum glucose metabolites have yet to show any significant patterns corresponding to brain injury or the thawed plasma response [24, 36]. Venturini et al. and others [37] suggested that substrate levels may also represent a new clinical target, citing trials that indicated that both high [38] and low parenchymal glucose (< 1 mmol/L) [33] corresponded to worse clinical outcomes, although others found no statistically significant relationship between outcomes and glucose levels [26]. This discrepancy may also reflect the impact of sample collection timing and catheter placement since proximity to lesion or “hyperglycolytic” regions may significantly alter metabolite levels/utilization [32].

**Table 1:** Glucose metabolism marker findings from select TBI metabolomics sets

Biomarker	Finding	Model	Reference
Glucose	- Increased ɑ-ketoglutaric acid in a biosignature at 4 days post injury predicts poorer GOSE outcomes at 12 months and predicts 30-day mortality	sTBI (Serum)	Banoei et al. [30]
	- Increased glucose, citrate & fumarate at 24 h post injury predict 3 month mortality		
	- Increasing levels of glucose, ribose & maltose predict increasing TBI severity	TBI (m/mod/s) (Serum)	Thomas et al. [31]
	- Increased glucose or mannose & sorbitol is associated with positive CT findings		
	- Increased levels of glucose, mannose, inositol & myoinositol predict unfavorable GOSE outcomes at 6 months		
	- Glucose derivatives, including 2,3-bisphosphoglyceric acid, are increased in sTBI patient sera (and to a lesser degree in mTBI patients) within 12 h after injury	TBI (m/mod/s) (Serum	Orešič et al. [34]
	- Increases in 4 unknown sugar derivatives predict unfavorable GOSE outcomes at 6–12 months		
	Increased sugar derivatives inositol, ribonic acid and 3-desoxy pentitol at 12 h after admission for TBI predicted positive CT findings	TBI (m/mod/s) (Serum)	Dickens et al. [41]
	- Increased Isovaleryl glucuronide is associated with mass lesion rather than diffuse lesion on CT		
	- Median CMD glucose 0–96 h after TBI is strongly correlated with total grey matter volume change, parietal grey matter volume change, cortical grey matter volume change and catheter region volume change (but not white matter volume change) over 12 months after injury	sTBI (CMD)	Bernini et al. 2023
	- CMD glucose was associated with outcomes when averaged over 7 days after injury	TBI (CMD)	Guilfoyle et al. [33]
	- Glucose was elevated from days 5–11 after injury in patients with unfavorable GOS outcomes relative to those with favorable outcomes		
	- Elevated CMD Glucose predicted mortality	TBI (m/mod/s) (CMD)	Timofeev et al. [26]
Lactate	- Increased lactate in a biosignature at 24 h and 4 days post injury predict poorer GOSE outcomes at 3 months	sTBI (Serum)	Banoei et al. [30]
	- Increased galactose is associated with positive CT findings		
	- Increased galacturonic acid predicts unfavorable GOSE outcomes at 6 months	TBI (m/mod/s) (Serum	Thomas et al. [31]
	- Median CMD LPR 0–96 h after TBI strongly correlates with total grey matter volume change (but not white matter volume change) over 12 months after injury		
	- No relationship between median CMD glutamate, pyruvate or lactate 0–96 h after TBI and brain volume changes observed	sTBI (CMD)	Bernini et al. 2023
	- CMD LPR was associated with outcomes when averaged over 72 h after injury	TBI (CMD)	Guilfoyle et al. [33]
	- Following an initial decrease during the first 48 h, LPR increases until day 14 after injury. LPR was elevated for about 10 days after injury in patients with unfavorable GOS outcomes relative to those with favorable outcomes		
	- Following an initial decrease during the first 48 h, Lactate increases until day 10 after injury. Lactate was elevated in patients with unfavorable outcomes relative to those with favorable outcomes		
	- CMD Lactate and LPR were elevated in patients who died and those with unfavorable GOS outcomes relative to those with favorable outcomes	TBI (m/mod/s) (CMD)	Timofeev et al. [26]
	- CMD LPR was elevated in pericontusional tissue relative to uninjured tissue		
	- Elevated CMD LPR predicted mortality		
Pyruvate	- Increased pyruvate at 24 h post injury predicts 3-month mortality	sTBI (Serum)	Banoei et al. [30]
	- Increased levels of pyruvic acid predict unfavorable GOSE outcomes at 6 months	TBI (m/mod/s) (Serum)	Thomas et al. [31]
	- Pyruvate gradually increases over 14 days after injury. Levels do not differ between unfavorable or favorable GOS outcomes	TBI (CMD)	Guilfoyle et al. [33]
	- Decreased CMD pyruvate predicted mortality	TBI (m/mod/s) (CMD)	Timofeev et al. [26]
Glutamate	- Reduced Glutamic acid was associated with “healthier” CMD state and was among the top 10 most relevant metabolites for Therapy Intensity Level (TIL) prediction	sTBI (CMD)	Eiden et al. [46]
	- Reduced Glutamic acid and increased glutamate in a biosignature at 24 h and 4 days post injury predict poorer GOSE outcomes at 3 months	sTBI (Serum)	Banoei et al. [30]
	- Decreased glutamine at 24 h post injury predicts 3-month mortality		
	- CMD Glutamate and glycerol were elevated in patients who died and those with unfavorable GOS outcomes relative to those with favorable outcomes	TBI (m/mod/s) (CMD)	Timofeev et al. [26]
	- Glutamate and glycerol trended higher in patients with unfavorable GOS outcomes relative to those with favorable outcomes	TBI (CMD)	Guilfoyle et al. [33]

CMD: Cerebral microdialysate; CT: Computed Tomography Scan; GOS(E): Glasgow Outcome Scale (Extended); LPR: Lactate Pyruvate Ratio; TBI: sTBI: severeTBI; modTBI: moderate TBI; mTBI: mild TBI); TIL: Therapy Intensity Level

### The lactate to pyruvate ratio

Stratification of metabolites for TBI most commonly relies on the lactate to pyruvate ratio (LPR) to capture the metabolic redox state [39]. Although serum sampling offers a broader picture of metabolism after TBI and lacks the specificity of cerebral microdialysis approaches, it clearly shows promise as a feasible enhancement to established models of prognostication based on clinical features. The addition of metabolic biomarkers improved the predictive ability of models using IMPACT and CRASH parameters to estimate severity/prognosis [18, 30, 31]. Serum-based analyses may also prove useful for early clinical decision making, most critically in determining the need for a head CT after TBI [40]. Dickens et al. identified several metabolites (including the sugar derivatives pentitol 3-desoxy, inositol, isovaleryl glucuronide, and ribonic acid) that outperformed the FDA-approved biomarkers GFAP/UCH-L1 in predicting positive CT findings [41]. It has been reported that serum levels of several sugar derivatives (e.g. inositol) may predict TBI outcomes and directly reflect levels within the cerebral parenchyma after injury [34].

Broadly, the literature consensus from cerebral microdialysate defines “metabolic crisis” as a post-TBI elevation in LPR beyond a certain threshold (Table 1) [28, 42]. A highly elevated LPR is associated with more unfavorable outcomes several months after injury and increased mortality in several clinical groups [26, 28, 33, 42]. A recent trial reported a significant association between elevated LPR during the first 96 h after injury and increased gray matter volume loss at 12 months after TBI (Table 1) [43]. Substrate levels can be used to further distinguish metabolic “fingerprints” corresponding to ischemic and anaerobic conditions (with elevated LPR in the absence of substrate/pO2) or mitochondrial dysfunction (elevated LPR despite normal substrate levels/perfusion). Evidence for these categories varies widely; however, a lower LPR (elevated pyruvate and lower lactate levels) was found to be protective [26, 43]. In general, glycemic control is an accepted concept in TBI management [27, 38], and supplementation with additional substrates such as lactate (intravenously or via CMD) may also show some clinical utility in reducing complications, like intracranial hypertension [44], when metabolic targets are better defined.

### Lipids, fatty acids, and ketogenic amino acids

In addition to the glucose metabolites explored above, several clinical trials and preclinical studies have revealed changes in lipid metabolism after TBI [30, 31]. Altered metabolites encompass a variety of lipid classes as well as metabolites associated with lipid metabolism, including fatty acids, phospholipids, ketone bodies, ketogenic amino acids (AAs), and branched-chain amino acids (BCAAs), which are derived from a variety of cellular and systemic compartments/processes [45]. A pilot study of cerebral dialysate from 26 ICU patients with TBI defined two clinically relevant metabolic states based on distinct changes in glucose, lactate and pyruvate as well as metabolites related to ketone metabolism [46]. The “healthier metabolic state” was defined by higher glucose metabolism substrate levels and corresponded to higher levels of ketogenic AAs/BCAA metabolite derivatives (tyrosine, threonine, valine, 4-methyl-2-oxovaleric acid and propionylcarnitine), ketone bodies (3-hydroxybutyrate and 2-hydroxybutyrate), and butyrylcarnitine (a metabolite involved in fatty acid metabolism; Table 2). This state was also associated with lower levels of the medium straight chain fatty acids, decanoic acid and octanoic acid (p = 0.06721) [46]. This study generated a metabolite model that could predict clinical outcomes, quantified by Therapy Intensity Level (TIL), in the initial and validation cohorts based on the levels of the ketogenic AA isoleucine, ketone bodies (β-hydroxybutyrate and acetoacetate), medium chain fatty acids (dodecanoic acid, suberic acid, 2-hydroxyoctanoic acid), medium chain fatty acid derivatives (10-hydroxydecanoic acid, 8-hydroxyoctanoic acid), and butyrylcarnitine (Table 2) [46]. Taken together, these findings indicate that TBI induces changes in key ketometabolic pathways, particularly fatty acid oxidation and ketogenic/BCAA metabolism [46]. However, the small cohort size contributed to underpowered statistical analysis and marked heterogeneity among patients, emphasizing the need for additional CMD trials in expanded cohorts.

**Table 2:** Lipid metabolism marker findings from select TBI metabolomics sets

Biomarker	Finding	Model	References
SMs	- Decreased SM 20:2 in a biosignature at 24 h and 4 days post injury predicting poorer GOSE outcomes at 3 months	sTBI (Serum)	Banoei et al. [30]
	- Increased SM 16:1 in a biosignature at 24 h post injury predicting poorer GOSE outcomes at 12 months		
	- Decreasing levels of SMs (d38:2, (40:2)/(18:1/22:1), d40:1) within 24 h after injury predict increasing TBI severity	TBI (m/mod/s) (Serum)	Thomas et al. [31]
	- Decreased SM(40:1) & SM(40:2), associated with positive CT findings		
	- Decreased SM (40:2)/(18:1/22:1), d40:1) predicts unfavorable GOSE outcomes at 6 months		
LPCs	- Increased LPCs (16:0, 17:0, 18:0) in a biosignature at 24 h post injury predicting poorer GOSE outcomes at 3 months	sTBI (Serum)	Banoei et al. [30]
	- Increased LPCs (14:0, 20:3 & 28:1) in a biosignature at 4 days post injury predicting poorer GOSE outcomes at 12 months		
	- Increased LPCs (28:1, 14:0) in a biosignature at 4 days post injury predicting poorer GOSE outcomes at 12 months		
	- Decreasing levels of LPCs (16:0, 18:0, 18:2, 20:5) within 24 h after injury predict increasing TBI severity	TBI (m/mod/s) (Serum)	Thomas et al. [31]
	- Decreased LPC(18:2) & LPC(20:5) associated with positive CT findings (Serum)		
	- Decreased LPC (18:0, 18:2, 20:5) predicts unfavorable GOSE outcomes at 6 months		
PCs	- Increased PC36:0 aa in a biosignature at 4 days post injury predicting poorer GOSE outcomes at 3 months	sTBI (Serum)	Banoei et al. [30]
	- Increased PCs (38:0 aa, 40:6 ae) and O-phosphocholine at 24 h post injury predict 3-month mortality		
	- Increased PCs (38:0 aa, 36:00 aa) at 4 days post injury predict 3-month mortality		
	- Decreasing levels of O-PCs (34:3, 36:3) within 24 h after injury predict increasing TBI severity	TBI (m/mod/s) (Serum)	Thomas et al. [31]
	- Decreased O-PC(34:2), O-PC(34:3), O-PC(36:3) associated with positive CT findings		
	- Decreased O-PC 3:3 predicts unfavorable GOSE outcomes at 6 months		
Ketogenic AAs/BCAAs	- Increased leucine, tyrosine, threonine, phenylalanine, valine, 4-methyl-2-oxovaleric acid & propionylcarnitine associated with “healthier” metabolic state	sTBI (CMD)	Eiden et al. [46]
	- Isoleucine among the top 10 most relevant metabolites for Therapy Intensity Level (TIL) prediction		
	- Decreased isoleucine, leucine, threonine phenylalanine & tyrosine in a biosignature at 24 h post injury predicting poorer GOSE outcomes at 3 months	sTBI (Serum)	Banoei et al. [30]
	- Increased valine, lysine, tyrosine, isoleucine & leucine in a biosignature at 4 days post injury predicting poorer GOSE outcomes at 3 months		
	- Increased tyrosine, leucine & valine at 24 h and 4 days post injury predicting poorer GOSE outcomes at 12 months		
	- Decreased BCAAs at 24 h post injury predict 3-month mortality		
	- Increased BCAAs, tyrosine, ornithine tryptophan & threonine at 4 days post injury predict 3 month mortality		
	- Threonine and BCAA breakdown products decrease with increasing TBI severity and their downregulation within 24 h after injury predicts positive CT findings		
	- AAs including threonine, tryptophan & phenylalanine are decreased in sTBI patient sera (but not in mTBI patients) within 12 h after injury	TBI (m/mod/s) (Serum)	Orešič et al. [34]
	- Decreased tryptophan and an unknown amino acid predict unfavorable GOSE outcomes at 6–12 (Serum) month		
	- Decreased serum 2-aminobutyric acid and an unknown amino acid within 12 h of admission for TBI predicted positive CT findings	TBI (m/mod/s) (Serum)	Dickens et al. [41]
Fatty acids	- Increased butyrylcarnitine associated with “healthier” metabolic state	sTBI (CMD)	Eiden et al. [46]
	- Decreased medium straight chain fatty acids (decanoic acid and octanoic acid) associated with “healthier” metabolic state		
	- Butryrylcarnitine, 10-hydroxydecanoic acid (10-OH-C10), suberic acid, 8-hydroxyoctanoic acid (8- OHC8), 2-hydroxyoctanoic acid, dodecanoic acid are among the top 10 most relevant metabolites for TIL prediction		
	- Increased propionic, stearic, octadecanoic, myristic, oleic and linoleic acid in a biosignature at 24 h post injury predicting poorer GOSE outcomes at 3 months	sTBI (Serum)	Banoei et al. [30]
	- Increased propionic acid, linoleic acid, valeric acid (C5) & medium-chain acylcarnitines in a biosignature at 24 h and 4 days post injury predicting poorer GOSE outcomes at 12 months		
	- Increased caproic acid (C6) & oleic acid in a biosignature at 4 days post injury predicting poorer GOSE outcomes at 12 months		
	- Increased acylcarnitines at 24 h post injury predict 3-month mortality		
	- Octanoic and decanoic acid levels within 24 h after injury increase with increasing TBI severity	TBI (m/mod/s) (Serum)	Thomas et al. [31]
	- Octanoic and decanoic acid are increased in sTBI patient sera (and to a lesser degree in mTBI patients) within 12 h after injury and predict unfavorable GOSE outcomes at 6–12 months	TBI (m/mod/s) (Serum)	Orešič et al. [34]
	- Increased decanoic acid is associated with mass lesion rather than diffuse lesion on CT	TBI (m/mod/s) (Serum)	Dickens et al. [41]
Ketone bodies	- Increased 3-hydroxybutyrate & 2-hydroxybutyrate associated with “healthier” metabolic state	sTBI (CMD)	Eiden et al. [46]
	- 3-hydroxybutyrate & acetoacetate among the top 10 most relevant metabolites for TIL prediction		
	- 3-hydroxybutyric acid & 2-hydroxybutyric acid are upregulated in sTBI patients (and to a lesser degree in mTBI patients) within 12 h after injury	TBI (m/mod/s) (Serum)	Orešič et al. [34]
	- Increased Acetoacetic acid (3-oxobutanoic acid) predicts poorer GOSE outcomes at 6–12 months		
	- Increased acetoacetic acid within 12 h of admission for TBI predicted positive CT findings	TBI (m/mod/s) (Serum)	Dickens et al. [41]
	- Increased 2-hydroxybutyric acid is associated with mass lesion rather than diffuse injury on CT		
NAA	- Increased NAA in a biosignature at 4 days post injury predicting poorer GOSE outcomes at 3 months	sTBI (Serum)	Banoei et al. [30]

CMD: Cerebral microdialysate; CT: Computed Tomography Scan; GOS(E): Glasgow Outcome Scale (Extended); LPCs = Lysophosphatidylcholine; NAA: N-Acetylaspartate; O-PCs: Ether-linked phosphatidylcholine; PC: Phosphatidylcholine; TBI: sTBI = severeTBI; modTBI: moderate TBI; mTBI: mild TBI); TIL: Therapy Intensity Level

Serum analyses of patients with TBI have also revealed changes in lipid metabolites, although these studies are less common than trials focused on glucose metabolism. Serum metabolomic studies, including some described in the prior sections, identified lipid metabolite clusters associated with TBI at 24 h [30, 31, 47] and 4 days [30] after injury using sera from TBI and control patients **(**Table 2). Several metabolites related to lipid metabolism ranked among the most robust metabolic markers for discrimination between moderate/severe TBI, mild TBI, and healthy controls (highest AUC, 0.665–0.9311) and were the best predictors of TBI outcomes according to logistic regression models [31]. These markers included four unidentified lipids, lysophosphatidylcholines (LPCs, 16:0, 18:0, 18:2, and 20:5), phosphatidylethanolamines (PEPs, P-18:0/22:5 and 20.1:20.4), cholesterol, an ether LPC (O-LPC 16:0), triglycerides (TGs, 51:20) and the ketogenic AA threonine [31]. The serum concentrations of LPCs, O-LPCs, several sphingomyelins (SMs, (40:2)/(18:1/22:1), d40:1), BCAAs and their breakdown products decreased in patients with severe TBI, whereas the levels of the medium chain fatty acids decanoic acid and octanoic acid increased with severity **(**Table 2) [31]. This finding aligns with prior research in which the levels of decanoic acid, octanoic acid, and 2- and 3-hydroxybutyric acids were increased in the sera and cerebral dialysate of patients with sTBI [34]. Reductions in the levels of serum BCAAs and their catabolic derivatives, including several acylcarnitines, were also associated with increasing TBI severity and predicted elevated ICP in a targeted analysis of these AAs [47]. Interestingly, severe TBI was also associated with lower BCAA levels than was orthopedic injury [47]. The detection of these signatures 24 h after TBI was associated with poorer clinical outcomes at 6 months [31].

Thomas et al. generated lipid metabolite clusters corresponding to CT pathological findings (i.e., mass lesions, intraventricular hemorrhage, etc.), which revealed consistent downregulation of threonine, LPC (18:2 and 20:5), O-LPC (34:2, 34:3 and 36:3) and SM (40:1 and 40:2) in the presence of positive imaging findings. Dickens et al. identified six serum metabolites that could specifically predict the need for imaging and reported that reductions in 2-aminobutyric acid (generated in the biosynthesis of the BCAA isoleucine) and increases in the ketone body acetoacetic acid corresponded to positive CT findings [41]. Additionally, higher levels of the fatty acids 2-hydroxybutyric acid and decanoic acid, isovaleryl glucuronide (related to glycogen synthesis) and a phenolic compound (originating from gut metabolism or exogenous propofol) were associated with CT mass lesions rather than diffuse injury [41] (Table 2). Additional studies examining the utility of these metabolites as biomarkers for clinical decision making should assess whether metabolite analysis can further enhance diagnosis as well as prognosis and the risk for complication management. Banoei et al. conducted similar analyses: they detected 188 total metabolites in serum collected 1 and 4 days after severe TBI using ^1^H-NMR and mass spectrometry and identified metabolite signatures that predicted favorable/unfavorable outcomes at 3 and 12 months after injury [30]. Increased LPCs (16:0, 17:0, 18:0), fatty acids (propionic, stearic, octadecanoic, myristic, oleic and linoleic acid), and decreased ketogenic AAs (isoleucine, leucine, threonine phenylalanine, tyrosine), and SM (20:2) at 24 h after admission predicted unfavorable outcomes at 3 months (Table 2) [30]. This finding aligns with the association of unfavorable outcomes with increased fatty acid metabolites and reduced ketogenic/BCAA levels reported in Banoei et al. [31] but contradicts Thomas et al.’s finding that increased LPCs and SM corresponded to more favorable outcomes at 6 months [31]. At four days postinjury, patients predicted to die had increased BCAA, ketogenic amino acid, fatty acid, and phosphatidylcholine (PC, 38:0 aa and 36:0 aa) levels and decreased acylcarnitine and LPC 26:0 levels [30]. Most predictive of poor clinical outcomes at 3 and 12 months were increases in LPCs (14:0, 16:0, 16:1, 17:0, 18:0, 18:2, 20:3 and 28:1), ketogenic AA, acylcarnitines, as well as BCAAs, NAA (n-acetylaspartate upregulates lipid degradation and fatty acid mobilization [48]), and tyrosine over the first four days after injury. Several studies chose to validate their defined signatures in a secondary dataset and noted improved prediction of outcomes when metabolic biomarkers were used with protein biomarkers (S100b, UCH-L1, GFAP, etc.) [30, 31]. Again, the metabolites identified above do not necessarily reflect the brain’s cellular processes in isolation, though BBB disruption, limited glymphatic [49] and passive transport to the periphery all increase after TBI [45]. A small trial compared the serum/blood levels of metabolites in arterial and jugular venous blood to indirectly assess metabolite uptake into the brain [50]. In addition to a net cerebral uptake of glucose-6-phosphate and depletion of systemic AAs, they noted a release of xanthine and choline. Their analysis also emphasized that some medications administered in ICU care may distort systemic metabolites or cerebral uptake and that these effects should be considered in the interpretation of clinical data [50].

Taken together, lipid pathways have not been as consistently described in clinical studies, as reflected in the limited pool of CMD/serum studies assessing lipid-associated metabolites in TBI. A recent review encompassing clinical metabolomic data emphasized the recurrence of key lipid biomarkers in sTBI: increased medium chain fatty acids (octanoic and decanoic acid) and choline as well as decreased BCAAs (valine, leucine) and NAA [51]. These signatures correspond to fatty acid metabolism, ketone and ketogenic/BCAA metabolism pathways. The clinical sites, sample sources (CENTER TBI [31, 41] and the CanTBI bank [30]), severity of TBI, health status of controls, time points and methods for characterizing lipid metabolism varied between trials and contributed to the observed differences. Even when the same experimental approach was used, findings in metabolomic studies were not always as consistent in the validation cohorts [41]. Expanding the amount and diversity of lipidomic data for patients with TBI will be necessary before changes in lipid metabolism can be considered reliable clinical biomarkers.

## New insight into glucose metabolism dysregulation in TBI

Current consensus suggests that primary TBI immediately resulting from mechanical stress and displacement of brain tissues is compounded by progressive secondary injury (damage to the BBB, protein aggregation, etc.) that is partially mediated by metabolic dysfunction [27]. Whether alterations in metabolism drive secondary injury processes or simply result from the subsequent depletion of cellular energy stores and oxidative stress is less clear. Studies suggest that these processes feed each other, leading to a cycle of damage in which initial metabolic disturbances are exacerbated by tissue destruction and vice versa [33, 52]. Characterizations of this state of “metabolic crisis” have distinguished low “fuel” (ischemic) states from conditions in which metabolic substrates are available but not effectively utilized (mitochondrial dysfunction). Critically, these states may require different approaches for clinical management and prognostication [38, 53, 54]. Consequently, distinguishing the mechanisms/processes underlying these distinct states may be highly informative for patient care.

Under normal conditions, the adult brain consumes more than 20% of metabolized glucose. The majority of this glucose is aerobically metabolized by astrocytes and, to a lesser extent, by oligodendrocytes, which ultimately release the metabolites (chiefly lactate) that fuel neurons though monocarboxylate transporters in glial-neuronal gap junctions [55]. This astrocyte–neuron lactate shuttle (ANLS), initially hypothesized by Pellerin and Magistretti, holds that BBB cells take up blood glucose via astrocytic/endothelial GLUT1 transport, allowing cerebral lactate buffering by astrocytic glycogen stores [56]. Lactate metabolism is critical for homeostatic axonal maintenance [55], but neurons also participate in the direct uptake of glucose (via GLUT3), which they metabolize primarily through oxidative phosphorylation. Downstream metabolites of glucose/lactate are ultimately used to generate ATP (the principal energy source for CNS cells) or engage in metabolite signaling [57]. Through these mechanisms, as well as systemic glucose targeting to the brain, cerebral metabolism relies on alternative substrates (ketone metabolism) only when glucose availability is severely disrupted [58]. TBI-induced ischemia, due to reduced CBF or microvascular damage that impedes blood glucose delivery despite preserved CBF, causes increased utilization of all these energy sources, which subsequently stresses cellular glucose/glycogen/lactate stores within the first few hours after injury [39, 59, 60]. Simultaneously, insulin resistance, up- and downregulation of vessel GLUT and increased BBB permeability drive systemic hyperglycemia and reduced utilization of glucose at the cellular level [38, 58, 61]. Ischemic cells compensate for reduced oxygen delivery by increasing anaerobic glycolysis, leading to acidosis as well as glucose derivatives that promote signaling related to oxidative stress and inflammation [38, 57, 62]. This is reflected in the increases in the LPR and the ratio of lactate to bicarbonate observed via magnetic resonance spectroscopic imaging in rats subjected to TBI [63]. This state of “relative hyperglycolysis” is facilitated by a hypoxia-induced increase in neuronal GLUT3 [62] and glycolytic enzymes, including pyruvate kinase and hexokinase, the rate-limiting enzymes of glycolysis. Notably, lactate dehydrogenase expression and activity are also increased days after TBI, providing additional ATP through lactate fermentation [64]. Inhibiting glycolysis through hexokinase knockdown in preclinical models acutely attenuated microglial activation and proinflammatory cytokine release after ischemic injury [65]. Pharmacological inhibition of glycolysis with 2-deoxyglucose reduced inflammatory signaling, posttraumatic hyperexcitability and epileptiform activity [66, 67]. As edema in the brain resolves (~ 48–120 h to days after injury), glycolytic enzymes and GLUTs are downregulated [60, 64]. Mild TBI models either recovered quickly or upregulated glycolysis more gradually compared to more severe injuries, suggesting that metabolic regulation depends on the degree of brain damage [64]. The differences in the mechanism of injury and outcomes in mild TBI models still limit our understanding of metabolic changes in these states [62, 64].

**Fig. 1:**
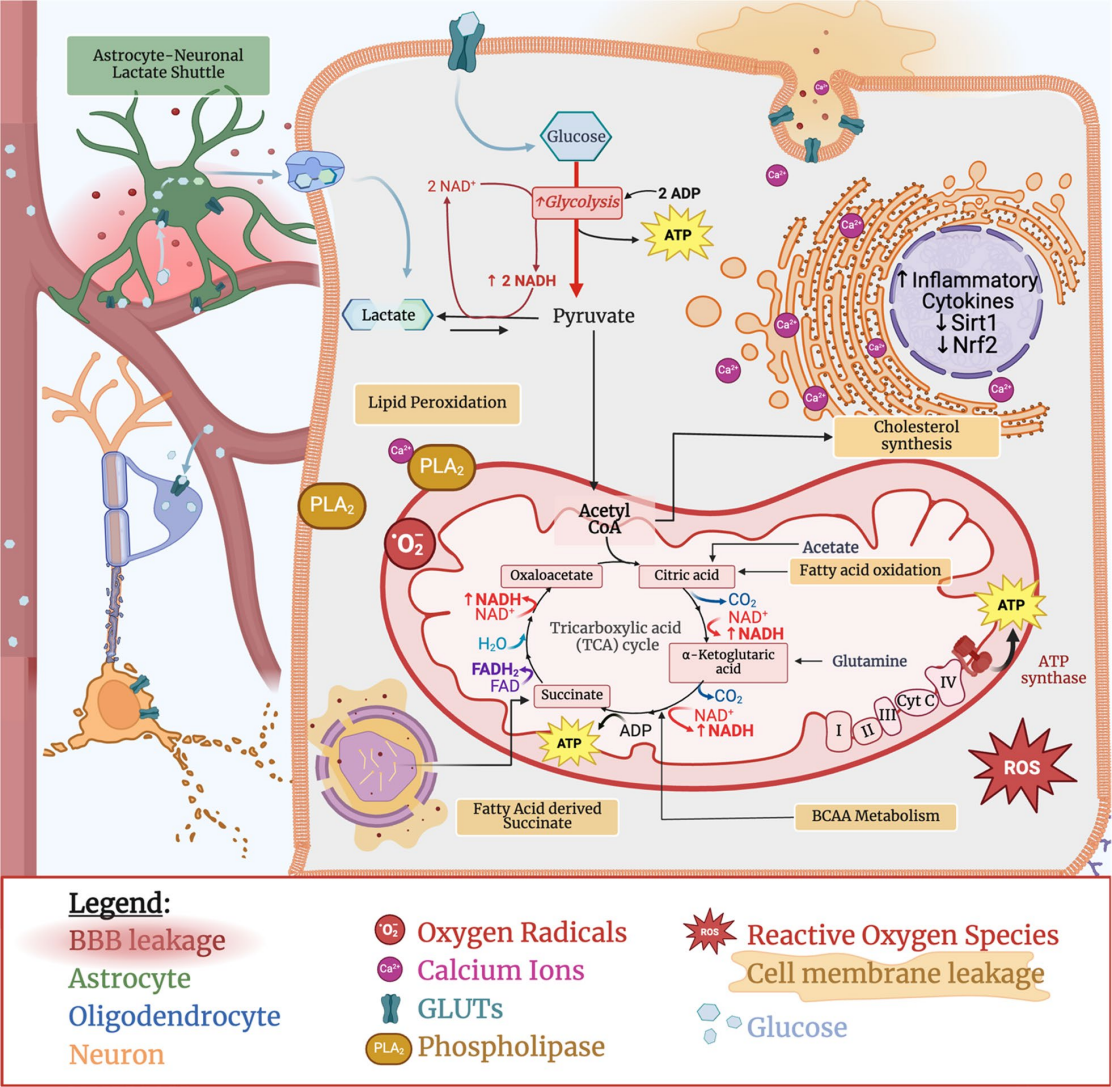
Cellular metabolism dysregulation in TBI. TBI-induced changes of canonical glucose metabolism including increased reliance on lactate and glycolytic metabolism, lipid derived metabolites, increased oxidative stress, inflammation and altered mitochondrial function are schematically displayed. ATP: Adenosine triphosphate; BBB: Blood Brain Barrier leakage; BCAA: Branched Chain Amino Acid; GLUTs: Glucose transporters; Cyt C: Cytochrome C; FAD: Flavin adenine dinucleotide; NAD: Nicotinamide adenine dinucleotide; Nrf2: Nuclear factor erythroid 2-related factor 2; PLA2: Phospholipase A2; ROS: Reactive Oxygen Species; Sirt1: Sirtuin 1

Since increased glycolysis, particularly in the context of impaired electron transport, increases the ratio of NADH to NAD +, excess NADH is thought to directly promote postischemic inflammation. Findings from other models of CNS degeneration, suggesting that normalization of the NADH/NAD + ratio promotes viability, support that this ratio (which is also a proxy for LPR) represents the connection between metabolic and inflammatory processes after injury [68, 69]. Protective treatment approaches aimed at restoring the NADH/NAD ratio also include increasing flux to parallel pathways such as the pentose phosphate pathway [57, 70], Krebs cycle (TCA) and oxidative phosphorylation (Fig. 1) [71, 72]. Studies with these approaches have revealed improved inflammation resolution and recovery through glutathione reactive oxygen species (ROS) and glycating agent scavenging [57], which are downregulated after severe injury [64]. Moreover, in lipopolysaccharide (LPS)-treated mice or primary microglial cultures, increasing NADH levels reversed the protective effects of glycolytic inhibition, driving NF-κB-mediated proinflammatory gene expression (IL1β, IL6, and iNOS) through dimerization of the NAD(H)-sensitive transcriptional corepressor CtBP [66]. It is well established that metabolic changes drive microglial polarization, and a number of pathways, including hypoxia-inducible factor-1alpha (HIF-1α), forkhead box P3 (FOXP3), and nuclear factor erythroid 2-related factor 2 (Nrf2) signaling, have been implicated in facilitating these effects [73].

## Impact of mitochondrial injury on metabolism dysregulation after TBI

Corresponding to an elevated LPR despite normal/elevated substrate levels, mitochondrial dysfunction is also common after TBI [28]. Impairments in the ability to generate ATP from canonical mitochondrial pathways, such as the TCA or electron transport chain, drive additional neurophysiological impairments even after primary injury and initial ischemia resolve. Clinical trials in patients with TBI have revealed reductions in cerebral glucose utilization years after injury, accompanied by persistent cognitive impairments [74]. This also prolongs the reliance on anaerobic sources of ATP: relative hyperglycolysis after TBI has been observed in the absence of hypoxia, when PbtO_2_ is normal [70]. Downstream of glycolysis, ATP generation through the mitochondrial TCA and electron transport is significantly reduced, even when substrate is in abundance [64, 72]. Ex vivo analysis of mitochondria derived from rats subjected to ballistic injury revealed decreases in the enzymatic activity of pyruvate dehydrogenase and glutamate dehydrogenase, which reduce input from glucose and glucogenic AAs into the citric acid cycle (reduced CoA-SH and acetyl-CoA concentrations), at 24 h after injury [72, 75]. Expression of these enzymes is also reduced but they are further inhibited by pyruvate dehydrogenase kinase isoforms, which are overexpressed in severe experimental TBI models [75]. Gene and protein expression of key TCA enzymes is also reduced in these models, while milder impact models actually demonstrate a gradual increase in these enzymes as mice recover from impact [75]. The reduced flux through the oxidative phosphorylation pathway also carries forward to the electron transport chain at the inner mitochondrial membrane. The activity of complex I and IV was reduced in isolated mitochondria after TBI [72]. The overall mitochondrial availability of NAD(H) and FAD(H) was reduced, reflecting a diminished capacity for electron and proton transport at the inner membrane. Posttraumatic leakage of electrons across the inner mitochondrial membrane drives free radical formation shortly after injury, which further damages cell membranes and nucleic acids.

After TBI, mitochondrial membrane disruption not only critically impairs oxidative phosphorylation at the site of electron transport but also activates downstream signaling pathways (ROS, caspases, calpains, etc.) that lead to cell death [76]. Uncoupling agents allow ions to move down their electrophysiological gradient, reversing the posttraumatic accumulation of protons (and Ca^2+^ ions) in the mitochondrial matrix that is thought to promote these signals [77]. For example, treatment with the 2,4-dinitrophenol prodrug MP201 restored maximal mitochondrial oxygen consumption as well as complex I respiration at 25 h in a mouse TBI model. Critically, MP201-treated mice exhibited improved novel object recognition, reduced cortical lesion volume and increased hippocampal neuron density two weeks after TBI [77]. Mitochondrial ion accumulation also contributes to the impact of antioxidant therapies [78, 79] that reduce the release/impact of mitochondrial ROS after TBI. The Nrf2 pathway is a key player in the antioxidant response to ROS accumulation that regulates glutathione peroxidase (GSH), heme oxygenase-1, NADPH oxidase (NOX) and several other critical pathways [80]. Nrf2 is also directly involved in maintaining mitochondrial membrane potential. After TBI, the NAD-dependent deacetylase sirtuin-1 (Sirt1) that promotes Nrf2 is downregulated, reducing Nrf2 signaling and Nrf2-induced transcription of antioxidant, antiapoptotic and metabolic genes [80]. Preventing this decrease, with the administration of a SIRT-1 activator, reversed ROS bursts, increased downstream nuclear PGC-1a and Nrf2 expression, and attenuated increases in mitochondrial membrane potential and neuronal apoptosis induced by ischemia/reperfusion injury [78]. Sirt1 activator treatment of neurons in vitro resulted in a dose-dependent increase in ATP, basal aerobic respiration, and pyruvate transport even in the presence of ETC inhibitors [78]. Pretreatment (but not treatment) also reduced infarct size and partially restored complex II, IV and V activity and complex I, II, and III expression [78]. Overall, dysfunctional mitochondrial respiration is linked to tissue damage and cognitive impairments observed after TBI, for which targeted therapies such as uncouplers and antioxidant therapies may be promising. Mitochondrial functions outside of canonical glucose metabolism are also critically disrupted by TBI, and key changes in lipid metabolism that result from TBI are further discussed in the following sections.

## Significance of lipid signals from clinical TBI data

Among the lipids implicated in clinical studies, several lipids and lipid metabolites are not directly utilized as fuel but nevertheless play an important role in TBI pathology [45]. Cell membrane phospholipids (ex. PC) may be released following mechanical damage to plasma membranes and BBB disruption after brain injury [45]. After the impact, the influx of Ca^2+^ ions into the cell cytoplasm leads to the phosphorylation/activation of phospholipases that cleave phospholipids into fatty acids, diaglycerols and lysophospholipids like LPC, which initiates signaling cascades that drive inflammation, BBB destruction, demyelination, and protein aggregation in several neurodegenerative contexts [81]. The principle lipase implicated in TBI is cytoplasmic phospholipase A2 (cPLA2), which is upregulated after in vivo and in vitro experimental TBI and preferentially cleaves arachidonic acid as an essential part of enzymatic lipid peroxidation and inflammatory signal transduction [82]. The activated cPLA2 complex also binds to the lysosomal membrane and facilitates the release of enzymes that damage organelle membranes, leading to mitochondrial damage, organelle degradation and cell death associated with secondary injury [82]. Other enzymes involved in peroxidation include cytochrome c/P450, lipoxygenases, and cyclooxygenases. Polyunsaturated fatty acids undergo nonenzymatic peroxidation when oxygen radicals generated by mitochondrial ROS production remove a hydrogen atom from the lipid, resulting in a lipid radical that can propagate to generate more peroxyl/alkoxyl radicals and hydroperoxide [83]. In spinal cord injury, this peroxidation has been shown to precede mitochondrial dysfunction [83], while inhibition of lipid peroxidation with 21-aminosteroid in rats attenuated controlled cortical impact (CCI)-induced decreases in mitochondrial respiration [84]. Administration of 21-aminosteroid attenuated post-CCI lipid peroxide accumulation and increased basal and maximal ETC activity as well as ETC/ATP synthase uncoupling [84]. Blocking lipid peroxidation also increased complex I and II activity and the overall efficiency of ETC coupling to ATP synthesis (as measured by the ratio of State III/IV O_2_ consumption: RCR). This finding suggested that lipid peroxidation contributes to the disruption of aerobic mitochondrial metabolism that defines secondary injury in TBI.

The relevance of lipid markers (SM, PC, cholesterol) to neuroinflammation-mediated reinjury of TBI brains is supported by the timeline of their release into the CSF both acutely and again at 4 days postinjury in patients [45]. Serial CSF collection from 10 patients with TBI revealed that patients who died exhibited a peak in CSF phospholipid levels, particularly PCs, on day 4 after injury [85]. Phospholipid release has been observed in experimental TBI models, which have shown an acute increase in serum SM and PC species as well as a decrease in serum TGs after mTBI in rats [86]. At subacute timepoints, these PCs and PEs (particularly those containing polyunsaturated fatty acids) as well as SMs and LPC 20:2 are downregulated, while diaglycerols and free fatty acids/polyunsaturated fatty acids are increased in serum of rats with TBI [87]. This suggests that an initial increase in phospholipid release is proportional to injury severity and is gradually reduced as these lipids are utilized (i.e., undergo peroxidation, oxidation, etc. [88]) (Fig. 1). Lower levels of phospholipids compared to the control group were maintained across all phospholipid species at 3 months and as late as 24 months after mild CCI, while lipid peroxide production increased [88]. Lipid oxidation into bioactive lipids, including oxidized cardiolipin, eicosanoids (prostaglandins, thromboxanes, leukotrienes, etc.), octadecanoids and docosanoids, also contributes to acute signaling changes after TBI [45, 89]. Although fatty acid oxidation produces pro- and anti-inflammatory signals immediately after injury, anti-inflammatory and vasodilatory signals persist in serum after proinflammatory octadecanoids and eicosanoids are cleared [89]. Despite promising preclinical data, targeting lipid peroxidation in clinical trials has not been successful [90], indicating that additional research exploring BBB penetrance of potential therapeutics and a better understanding of how lipid peroxides work in the temporal/biological context of TBI will be necessary before these metabolites can be harnessed for clinical benefit.

## Lipids as an energy source in TBI metabolism

In addition to the effects of lipid peroxides on mitochondrial membrane integrity and ETC activity, lipids may be used as alternative fuel sources in low glucose or aerobic metabolic states generated by TBI. Metabolic dysfunction induced by TBI impairs the availability/utilization of cerebral glucose for aerobic ATP generation and promotes release of alternative substrates (i.e. ketone bodies, KBs) to fuel the increased metabolism demanded for brain recovery. The brains’ ability to maintain a stable/optimal glucose level is associated with successful recovery in other CMD studies [28]. To accomplish this, glucose transport to the brain is increased [62] while increased cerebral reliance on KB metabolism reduces the need for glucose consumption. Bernini et al. [91] observe that brain KBs and blood glucose are inversely correlated such that a systemic drop in blood sugar (in part due to increased uptake into the brain), is associated with increased systemic production and cerebral uptake of KBs. Shifting this metabolic balance toward ketometabolism is thought to reduce the reliance on anaerobic metabolic processes that drive oxidative stress [92]. After TBI, increased expression of monocarboxylate transporters, which import ketone bodies generated by astrocytes or from fatty acids in the liver into neurons, facilitates increased ketometabolism [93]. Ketone bodies are converted by ketolytic enzymes to acetyl-CoA, which can enter the TCA for energy generation [93]. Fatty acid oxidation in cell peroxisomes also generates succinate, which can also enter this cycle and offers another path for lipids in cell metabolism [94] (Fig. 1). While the activity of ETC components decreases following TBI, evidence regarding the activity of complex II, which accepts electrons from ketones, varies and may not show as significant inhibition as other complexes [72, 84]. Several studies have demonstrated the therapeutic benefit of a ketogenic diet for recovery in adult rodent TBI models as well as in pediatric mice, which rely on ketone metabolism at baseline [95]. A high-fat, low-carbohydrate diet increases the serum ketone body concentration in mTBI mice and restores visual and spatial memory performance and reverses neuronal loss in the cortex and hippocampus [92]. Increasing fatty acid and ketone metabolism after a ketogenic diet also attenuated reductions in ATP, NAD, mitochondrial fragmentation, and myelination in a murine diffuse axonal injury model, while treatment with ketone bodies reduced neuronal/myelin damage induced by glucose deprivation in vitro [96]. A high-fat diet also increased Sirt1 expression up to one month after injury, suggesting that the Sirtuin pathway also contributes to improvements in mitochondrial metabolism and oxidative stress associated with following ketone supplementation [92]. Downstream changes may include ketogenic diet-induced increases in mitochondrial uncoupling protein expression and activity [97], which improve cognitive, pathological and mitochondrial recovery after experimental TBI [77]. Uncoupling may also contribute to the ETC activity changes observed in lipid peroxidation inhibition (since blocking this process frees up additional fatty acids for metabolism).

It should be noted that most preclinical works explore TBI ketometabolism through nutritional supplementation and do not directly assess endogenous ketones in TBI pathology. Perhaps due to these unknowns or the concerns about the effects of prolonged ketosis, ketogenic diets have not yet passed beyond phase I clinical trials for TBI [98]. While short term implementation of ketogenic diets has shown potential in several neurodegenerative diseases for improving metabolic function and producing neuroprotective and anti-inflammatory intermediates [99], long-term implementation is limited by decreased patient adherence and occasional adverse cardiac, renal and cognitive events (particularly in pediatric patients) [100]. Accordingly, there are very few studies examining the impact of long-term ketosis exceeding 24 or even 12 months. Ketogenic diets that induce greater ketosis also suffer pooper compliance due to side effects, diet restrictiveness and psychosocial factors compared to milder ketogenic diets [101]. Common early adverse effects after beginning ketogenic diet include mild headaches, digestive system complaints or wooziness [102]. Although far less common, long-term increases in free fatty acids and reduced short-chain fatty acid production can precipitate myocardial insulin-resistance [102] while ketone bodies can promote cardiac scarring and cell senescence [103]. Severe restriction has rarely been associated with cognitive/systemic changes induced by ketoacidosis [104, 105]. Physicians considering this intervention for patients with cardiac risk factors must consider the long term impact of increased nutritional fat content and low fiber diet, such as cardiovascular damage and renal complications like kidney stones [103]. Due to lack of research on prolonged ketosis in the context of TBI, the extent to which adverse events of ketogenic diets could impact the unique pathological state of these patients remains a concern for many physicians that recognize its clinical potential [106].

## TBI-induced changes of systemic and microbiome metabolism

In addition to the cellular pathways discussed here, TBI impacts systemic metabolism through systemic immune activation and by altering brain signaling that maintains homeostatic metabolic function [i.e. (para)sympathetic nervous system function & neuroendocrine signaling [107]]. Concurrently, shifts in GI function/microbiota feedback on the CNS through rapid neurotransmission by the autonomic nervous system and vagus nerve or more slowly through the release of cytokines, pathogen-associated molecular patterns, metabolites, hormones and even neurotransmitters [108]. These fluctuations directly contribute to the changes in serum metabolites and to cerebral metabolism that we have described here. As a consequence, interpretation of “omics” data must consider the bidirectional gut-brain connection which facilitates the delivery of metabolites to the CNS and impacts GI function/microbiota after brain injury [107]. Brain injury induces the release of inflammatory and stress signals which increase enteric glial cell activation, immune cell infiltration and mucosal barrier permeability, decrease GI motility and alter the populations of enteric microbiota [107]. Mechanical or inflammatory injury to the hypothalamus alters neuroendocrine secretion and contributes to systemic insulin resistance induced by inflammation/hyperglycemia, further impairing metabolic regulation and glucose uptake [60]. The external risk factors associated with TBI, such as antibiotic administration, hospital stay, enteral nutrition, etc., can also increase this risk [109, 110]. These clinical decisions correspond to exacerbation or attenuation of the TBI-induced reduction in GI microbiome diversity over time [109]. The extent of microbiome distortion is also associated with severity of injury: reductions in microbiome diversity and overgrowth of some bacterial phyla/species upon admission predicted mortality in trauma patients [111]. Moreover, TBI was associated with reduced commensal bacterial species and increased relative abundance of pathogenic bacteria in oral, rectal and skin samples from pediatric study participants [109]. In a preclinical TBI model, ablation of this progression through antibiotic depletion of gut microbiota attenuated CA-1 (but not CA-3) neuronal loss, microgliosis, and associative learning deficits induced by CCI [112]. Microbiome depletion also reduced cortical lesion size and the loss of cecal tight junction staining [112]. As gut microbiota are highly responsive to diet, and nutritional support is already a protocolized part of TBI care (due to observational studies demonstrating an association between delayed enteral nutrition and worse neurologic outcome [113]) there has been much interest in identifying dietary interventions which can improve recovery and reduce mortality [114]. In a 2013 review of smaller randomized control trials and non-randomized prospective studies, Wang et al. observed that better outcomes (mortality, infection/complication rate, functional outcome score) were associated with early refeeding, a combination of parenteral and enteral nutrition, and “immune enhancing” formulas supplemented with a combination of probiotics, glutamine, arginine, fats (saturated and poly unsaturated, omega-3 fatty acids), protein, vitamins and minerals [114–117]. However, these interventions have not been tested in a robust, large scale randomized trial and conclusions from prior assessment are often contradictory [118]. These trials have not explored the impact of this supplementation on the gut microbiome but have demonstrated beneficial effects of parenteral lactate [119–121] and oral [122, 123] or parenteral BCAAs [124, 125]. Other supplements, such as omega-3 fatty acids, short chain fatty acids, and ketogenic diets, that have generated results in animal models, have yet to be tested in clinic [92, 126, 127]. The benefits of ketogenic diets described above, for instance, demonstrate the importance of GI microbiota, which is a required intermediary for the effect of these diets in seizure protection (by modulating GABA production or AMPA receptor binding, for instance [128, 129]). Probiotic therapies are also being applied with various degrees of success in a broad spectrum of neurodegenerative diseases [130]. These approaches are subject to many of the same limitations as nutritional/microbiota therapies for TBI, including the lack of a suitable animal model of the human microbiome, heterogeneity in the methods/quality of microbiome data collection, the protracted timeline and inevitable presence of confounding variables in human diet trials [130].

## Conclusion

TBI drives changes to cerebral and systemic metabolism that reduce aerobic metabolism of glucose in the brain and compensate by upregulating alternative pathways such as anaerobic glycolysis, lactate fermentation, gluco/ketogenic amino acid metabolism, and lipid metabolism. Here, we discussed relevant changes to these pathways and their clinical utility in predicting imaging findings, mortality and functional outcomes. Preclinical study of the metabolic pathway disruptions that give rise to these biomarkers facilitates enhanced understanding of TBI pathology and identification of new therapeutic targets. However, clinical trials investigating these pathways, are subject to limitations including the heterogeneity in TBI severity/etiology, variations in experimental time points and the methods applied to measure metabolites. Mass spectrometry or ^1^H-NMR-based capture of metabolites in cerebral microdialysate is limited by the invasiveness of CMD catheter placement and the regional specificity of detected parenchymal changes [131]. In vivo PET, MRI or CT scanning are more easily administered but do not reflect temporal changes to metabolism unless administered at greater frequency than clinically indicated [131]. Studies of serum metabolites allows for larger cohort sizes and ease of implementation but is an indirect marker of cerebral metabolism that is impacted by GI function/microbial metabolism. Nevertheless, therapeutic interventions aimed at altering cerebral or systemic metabolism have been promising. This review emphasizes the need for additional research into the metabolic underpinnings of TBI pathology, particularly into emerging areas like lipid and microbiome driven processes.

## Data Availability

Data sharing is not applicable to this article as no datasets were generated or analysed during the current study.

## Abbreviations

2,3-BPG: 2,3-Bisphosphoglyceric acid
*AA*: Amino acid
ANLS: Astrocyte-neuron lactate shuttle
ATP: Adenosine triphosphate
AUC: Area under the curve
BBB: Blood–brain barrier
BCAA: Branched chain amino acid
CCI: Controlled cortical impact
CMD: Cerebral microdialysis
CNS: Central nervous system
coA: Coenzyme A
*cPLA2*: Cytosolic phospholipases A2
*CSF*: Cerebrospinal fluid
*CT*: Computed tomography
ETC: Electron transport chain
FOXP3: Forkhead box P3
GCS: Glasgow coma scale
GFAP: Glial fibrillary acidic protein
GLUT: Glucose transporters
GOS(E): Glasgow outcome scale (extended)
GSH: Glutathione peroxidase
H-NMR: Proton nuclear magnetic resonance
HIF-1α: Hypoxia-inducible factor-1alpha
ICP: Intracranial pressure
ICU: Intensive care unit
IL: Interleukin
iNOS: Inducible nitric oxide synthase
LPC: Lysophosphatidylcholine
LPR: Lactate/Pyruvate ratio
LPS: Lipopolysaccharide
MS: Mass spectrometry
NAA: N-acetylaspartate
NAD(H): Nicotinamide adenine dinucleotide
NADP(H): Nicotinamide adenine dinucleotide phosphate
NF-κB: Nuclear factor kappa B
NOX: NADPH oxidase
NRF2: Nuclear factor erythroid 2-related factor 2
O-LPC: Ether lysophosphatidylcholine
PbtO2: Partial pressure of brain tissue oxygen
PEP: Phosphatidylethanolamines
PET: Positron emission tomography
PGC1a: Peroxisome proliferator–activated receptor gamma coactivator-1 alpha
ROS: Reactive oxygen species
S100B: S100 calcium-binding protein B
Sirt1: NAD-dependent deacetylase sirtuin-1
SM: Sphingomyelin
TBI: Traumatic brain injury
TCA: Citric acid cycle, krebs cycle
TG: Triglyceride
TIL: Therapeutic intensity level
UCH-L1: Ubiquitin C-terminal hydrolase-L1
